# Risk of Acute Myocardial Infarction in Patients with Gastroenteritis: A Nationwide Case-Control Study

**DOI:** 10.3390/jcm11051341

**Published:** 2022-02-28

**Authors:** Ying-Hsuan Tai, Ming-Long Chang, Pao-Hsien Chu, Chun-Chieh Yeh, Yih-Giun Cherng, Ta-Liang Chen, Chien-Chang Liao

**Affiliations:** 1 Department of Anesthesiology, Shuang Ho Hospital, Taipei Medical University, New Taipei City 235, Taiwan; tp16960@gmail.com (Y.-H.T.); stainless@s.tmu.edu.tw (Y.-G.C.); 2 Department of Anesthesiology, School of Medicine, College of Medicine, Taipei Medical University, Taipei 110, Taiwan; tlc@tmu.edu.tw; 3 Department of Emergency Medicine, Taipei Medical University Hospital, Taipei 110, Taiwan; shaulongking@gmail.com; 4 Department of Emergency Medicine, School of Medicine, College of Medicine, Taipei Medical University, Taipei 110, Taiwan; 5 Division of Cardiology, Department of Internal Medicine, Linkou Chang Gung Memorial Hospital, Taoyuan 333, Taiwan; taipei.chu@gmail.com; 6 Department of Surgery, China Medical University Hospital, Taichung 404, Taiwan; b8202034@gmail.com; 7 Department of Surgery, University of Illinois, Chicago, IL 60637, USA; 8 Department of Anesthesiology, Wan Fang Hospital, Taipei Medical University, Taipei 116, Taiwan; 9 Anesthesiology and Health Policy Research Center, Taipei Medical University Hospital, Taipei 110, Taiwan; 10 Department of Anesthesiology, Taipei Medical University Hospital, Taipei 110, Taiwan; 11 Research Center of Big Data and Meta-Analysis, Wan Fang Hospital, Taipei Medical University, Taipei 116, Taiwan; 12 School of Chinese Medicine, College of Chinese Medicine, China Medical University, Taichung 404, Taiwan

**Keywords:** gastroenteritis, dehydration, acute myocardial infarction, case-control study, hospitalization

## Abstract

Gastroenteritis promotes the development of systemic inflammation and a hypercoagulable state. There are limited data regarding the association between gastroenteritis and acute myocardial infarction (AMI). We aimed to evaluate the risk of AMI after an episode of gastroenteritis. In this nested case-control study, we selected patients who were hospitalized for AMI (*N* = 103,584) as a case group during 2010–2017 and performed propensity score matching (case-control ratio 1:1) to select eligible controls from insurance research data in Taiwan. We applied multivariable logistic regressions to calculate adjusted odds ratios (ORs) with 95% confidence intervals (CIs) for the risk of AMI associated with recent gastroenteritis within 14 days before AMI. We also compared the outcomes after AMI in patients with or without gastroenteritis. A total of 1381 patients (1.3%) with AMI had a prior episode of gastroenteritis compared to 829 (0.8%) among the controls. Gastroenteritis was significantly associated with a subsequent risk of AMI (adjusted OR: 1.68, 95% CI: 1.54–1.83), which was augmented in hospitalizations for gastroenteritis (adjusted OR: 2.50, 95% CI: 1.20–5.21). The outcomes after AMI were worse in patients with gastroenteritis than in those without gastroenteritis, including increased 30-day in-hospital mortality (adjusted OR: 1.28, 95% CI: 1.08–1.52), medical expenditure, and length of hospital stay. Gastroenteritis may act as a trigger for AMI and correlates with worse post-AMI outcomes. Strategies of aggressive hydration and/or increased antithrombotic therapies for this susceptible population should be further developed.

## 1. Introduction

Ischemic heart disease remains a leading cause of mortality and morbidity, accounting for approximately 10 million annual cases of myocardial infarction and causing a substantial public health burden worldwide [1,2,3]. Acute myocardial infarction (AMI) is triggered by a variety of factors, including physical exertion [4,5], emotional stress [4,5], respiratory infection [6,7,8,9,10,11,12], and a hypercoagulable state [13]. The hypercoagulable state predisposes patients to either experience atherothrombotic events relatively early in life or suffer from recurring events throughout their lifespan [13]. Accumulating evidence has shown that the inflammatory response may directly affect coagulation pathways and contribute to a hypercoagulable state [13,14].

Gastroenteritis has also caused a global epidemic, affecting approximately 6 billion people and leading to 1.5 million deaths globally in 2017 [1,2]. Inflammation of the gastrointestinal tract may cause diarrhea and dehydration, which further result in hemoconcentration and elevated blood viscosity [15,16]. Severe dehydration predisposes patients to venous thromboembolism, which has been reported in those with ischemic stroke and marathon athletes [17,18]. Gastroenteritis promotes the development of systemic inflammation and a hypercoagulable state; these complications may contribute to the pathogenesis of coronary artery thrombosis [13,14]. An epidemiological survey showed that bacterial gastroenteritis may elevate the risk of hypertension and cardiovascular diseases [19]. However, the previous study has some limitations, such as a small sample size and self-reported cardiovascular diseases [19]. Limited information is available on the association between gastroenteritis and the risk of AMI.

To clarify the relationship between gastroenteritis and myocardial infarction, we conducted this nested case-control study based on a nationwide sample to evaluate the risk of developing AMI after an episode of gastroenteritis. We also investigated the possibility of gastroenteritis influencing worse postmyocardial infarction outcomes.

## 2. Methods

### 2.1. Source of Data

We conducted this study by utilizing research data from the National Health Insurance in Taiwan. The insurance program was implemented in March 1995 and covers more than 99% of the local population and foreigners working or studying in Taiwan. A detailed description of the research database was given in our previous studies [20,21]. Informed consent was not required because our research data were based on deidentified information. This study was approved by the institutional review board of Taipei Medical University (TMU-JIRB202101004).

### 2.2. Study Design

**Figure 1:**
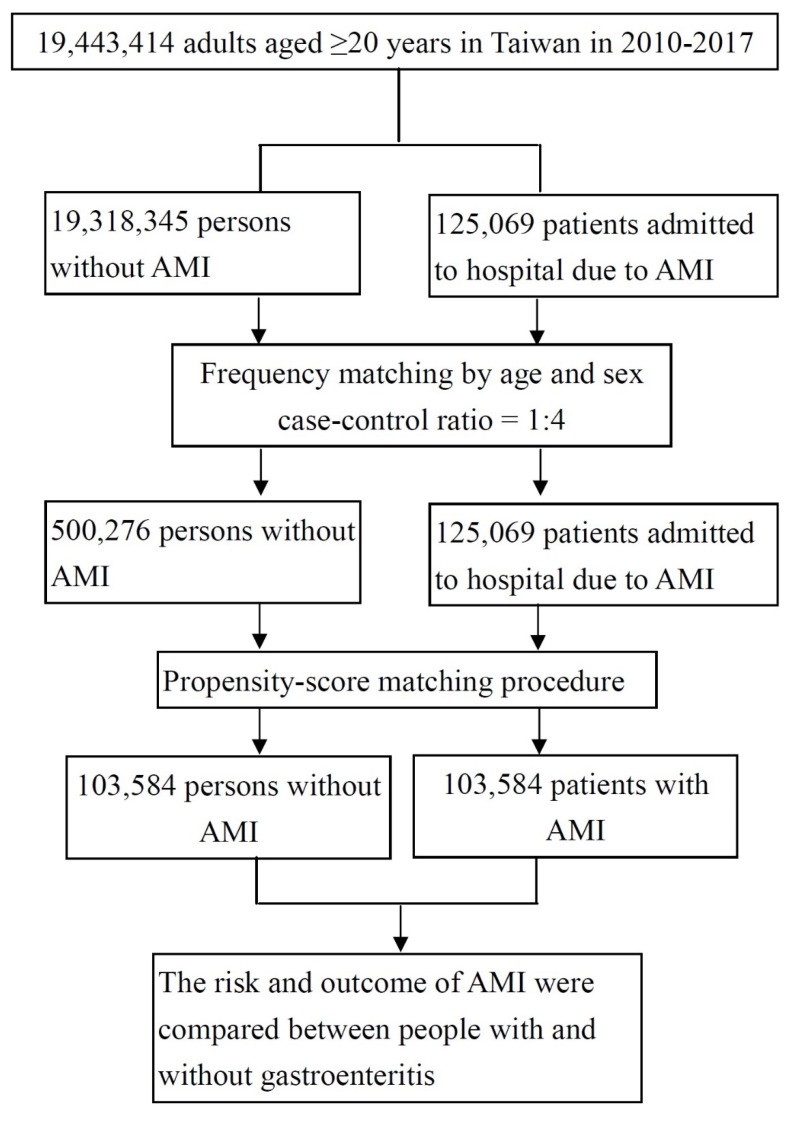
The process of selection of study subjects.

From the database of Taiwan’s National Health insurance which included the medical information of 19,443,414 adults aged 20 years and above in 2010–2017, we identified 125,069 patients who were admitted to the hospital due to AMI as the case group in this case-control study. For the control group, we firstly used the frequency matching by age and sex (case-control ratio = 1:4) to select 500,276 eligible people without AMI history (Figure 1). In the case group, patients with any diagnosis of AMI within the previous two years were excluded to ensure that all AMI cases were first diagnosed with AMI. For each AMI case, one control without a history of AMI was randomly selected from the same index year. Random selection of controls allows us to distinguish differences between AMI cases and general insured adults. We especially considered the potential confounding factors that might influence the association between gastroenteritis and AMI. For selecting eligible cases and controls for comparison, we performed propensity score matching (case-control ratio 1:1) and selected 103,584 patients with AMI hospitalization as final case group and 103,584 adults who had no AMI during the same period as the control group. Among cases and controls, people who had been diagnosed with gastroenteritis within the last 14 days before the index date were considered the main exposure in this study. Those who visited outpatient, emergency, and inpatient medical care with a physician’s diagnosis of gastroenteritis were eligible patients with gastroenteritis. Our purpose was to investigate whether recent gastroenteritis is a risk factor for AMI.

In further analyses investigating the effects of gastroenteritis on post-AMI outcome, we compared the 30-day in-hospital mortality, intensive care, length of hospital stay, and medical expenditure between AMI patients with and without gastroenteritis.

### 2.3. Definition and Measures

Sociodemographic variables used in this study included sex, age, and low-income status. Low-income status was defined as having a low income within 2 years before the index date. The details of the definition of low income were described in previous studies [20,21]. We utilized the International Classification of Diseases, Ninth Revision, Clinical Modification (ICD-9-CM) and physicians’ diagnoses to identify main exposure, gastroenteritis (558.2, 558.3, 558.9), AMI (410), and coexisting medical conditions within the previous 24 months [20,21], such as hypertension (401–405), diabetes (250), ischemic heart disease (410–414), mental disorders (290–319), chronic obstructive pulmonary disease (491, 492, 496), hyperlipidemia (272.0, 272.1, 272.2), heart failure (428), stroke (430–438), liver cirrhosis (571), and atrial fibrillation (427.31). We implemented administration codes (D8 and D9) to identify renal dialysis in the research database of Taiwan’s National Health Insurance. Recent visits for emergency care and hospitalization were also considered potential confounding factors.

### 2.4. Statistical Analyses

In this study, we used a propensity-score matching procedure to balance the potential confounding factors between patients with AMI and non-AMI controls. We developed a nonparsimonious multivariable logistic regression model to estimate a propensity score for patients with AMI. Clinical significance guided the initial choice of covariates in this model: age, sex, low income, hypertension, diabetes, ischemic heart disease, mental disorders, chronic obstructive pulmonary disease, hyperlipidemia, heart failure, stroke, renal dialysis, liver cirrhosis, and atrial fibrillation. We matched patients with AMI to non-AMI controls using a greedy matching algorithm (without replacement) with a caliper width of 0.2 SD of the log odds of the estimated propensity score. This method could remove 98% of the bias from measured covariates.

We utilized chi-square tests with summarizing frequency (percentage) to compare the categorical data regarding sociodemographic factors, medical conditions, and infection of gastroenteritis between cases with AMI and controls. Multilevel logistic regression analysis was used to estimate the odds ratios (ORs) and 95% confidence intervals (CIs) of AMI associated with gastroenteritis after controlling for potential confounding factors. In further analyses, we calculated the adjusted ORs and 95% CIs of post-AMI intensive care and 30-day in-hospital mortality associated with recent gastroenteritis. The length of hospital stay and medical index admission for AMI were also compared by summarizing the mean (standard deviation) between patients with and without recent gastroenteritis. All analyses were performed with the statistical package SAS for Windows (Version 9.1, SAS Institute Inc., Cary, NC, USA). A *p* value < 0.05 was considered statistically significant. All tests were two-tailed.

## 3. Results

Table S1 shows the baseline characteristics of patients with AMI and non-AMI controls under the frequency matching. After propensity score matching (Table 1), there were no significant differences between the two groups in age, sex, low income, emergency care, inpatient care, hypertension, diabetes, ischemic heart disease, mental disorders, chronic obstructive pulmonary disease, hyperlipidemia, heart failure, stroke, renal dialysis, liver cirrhosis, or atrial fibrillation. Among the 103,584 AMI cases and 103,584 controls, cases had a higher prevalence of gastroenteritis than controls (1.3% vs. 0.8%, *p* < 0.0001).

In Table 2, patients with recent gastroenteritis had a higher risk of AMI than those with no gastroenteritis (OR 1.68, 95% CI 1.54–1.83). The further sensitivity analysis (Table S2) also showed that gastroenteritis was associated with AMI (OR 2.89, 95% CI 2.13–3.92). Recent gastroenteritis infection was associated with AMI in men (OR 1.56, 95% CI 1.40–1.72), women (OR 2.01, 95% CI 1.71–2.36), and people aged 20–49 years (OR 1.50, 95% CI 1.17–1.93), 50–59 years (OR 2.09, 95% CI 1.68–2.61), 60–69 years (OR 1.49, 95% CI 1.25–1.79), 70–79 years (OR 1.53, 95% CI 1.28–1.82), and ≥80 years (OR 1.86, 95% CI 1.57–2.22). The adjusted ORs of AMI associated with gastroenteritis for people with 0, 1, 2, and ≥3 medical conditions were 1.76 (95% CI 1.45–2.14), 1.78 (95% CI 1.53–2.06), 1.77 (95% CI 1.49–2.10), and 1.34 (95% CI 1.10–1.62), respectively. An association between gastroenteritis and the risk of AMI existed in people with a history of stroke (OR 2.15, 95% CI 1.22–3.78) and ischemic heart disease (OR 1.27, 95% CI 1.07–1.50). In further analyses, (Table 3), patients with recent hospitalization for gastroenteritis had an increased risk of AMI compared with those who did not (OR 2.50, 95% CI 1.20–5.21).

Among 103,584 patients hospitalized for AMI (Table 4), 1726 had recent infection of gastroenteritis before AMI. Compared with patients without gastroenteritis, patients with gastroenteritis had higher proportions of females (*p* < 0.0001), people older than 80 years (*p* < 0.0001), low income (*p* < 0.0001), ≥3 hospitalizations (*p* < 0.0001), and ≥3 emergency visits (*p* < 0.0001). Higher proportions of hypertension (*p* < 0.0001), diabetes (*p* = 0.0002), mental disorders (*p* < 0.0001), chronic obstructive pulmonary disease (*p* = 0.0005), heart failure (*p* = 0.0020), hyperlipidemia (*p* = 0.0083), and liver cirrhosis (*p* = 0.0469) were found in patients with gastroenteritis than in those without gastroenteritis.

Compared to patients without gastroenteritis (Table 5), patients with gastroenteritis had a higher risk of post-AMI mortality (OR 1.28, 95% CI 1.08–1.52). Medical expenditures (5905 ± 6511 vs. 5531 ± 5115, *p* = 0.0174) and hospital length of stay (8.7 ± 8.6 vs. 7.7 ± 8.1 days, *p* < 0.0001) during the admission of AMI were comparatively greater for patients with gastroenteritis.

## 4. Discussion

This study demonstrated that gastroenteritis was significantly associated with a subsequent risk of AMI, which was further augmented in patients with gastroenteritis requiring hospitalizations. In addition, the outcomes after AMI were worse in patients with gastroenteritis than in those without gastroenteritis, including increased in-hospital mortality, medical expenditure, and length of hospital stay. This association was independent of age, sex, and other cardiovascular risk factors. These results provide important implications for preventing the occurrence of myocardial infarction in patients at high cardiovascular risk.

Our study is the first to demonstrate a higher risk of AMI associated with gastroenteritis. The relationships between respiratory tract infections and AMI risk have been extensively investigated in prior studies, such as influenza and pneumonia [6,7,8,9,10,11,12]. However, no study has focused on the association between gastrointestinal inflammation or infection and AMI risk. A recent study showed that various types of inpatient infections were strong triggers for coronary heart disease, including digestive infections [11]. However, the investigators did not reveal the results of subgroup analysis pertinent to digestive infections [11]. Another study analyzed the incidence of cardiovascular diseases after a regional outbreak of *Escherichia coli* O157:H7 gastroenteritis and showed no increase in the risk of cardiovascular events or death [22]. However, this study has the limitations of a small patient sample and a fairly long time frame, which makes it difficult to accurately clarify the relationship between gastroenteritis and AMI risk [22]. Our results showed that 1.3% of AMI hospitalizations were concomitant with a prior episode of gastroenteritis and linked to increased mortality risk and length of hospital stay. Whether gastroenteritis acts as a predisposing or precipitating factor for myocardial infarction is unclear, which warrants future studies to elucidate this question.

Several possible mechanisms may be helpful for explaining the higher risk of AMI in patients with recent gastroenteritis. First, gastroenteritis-related diarrhea may cause dehydration or hypovolemia and hemoconcentration afterward [15,16]. Elevated whole-blood and plasma viscosity is a recognized risk factor for AMI and can be induced by dehydration [23]. In patients with AMI, high plasma viscosity on admission was linked to an increased risk of shock, thromboembolism, and left ventricular failure [24]. Second, hypernatremia caused by diarrhea stimulates the production of the clotting initiator, von Willebrand factor, in endothelial cells and promotes thrombogenesis [25,26]. In animal experiments, elevation of extracellular sodium induced by water restriction upregulates the gene expression of vascular cell adhesion molecule 1, E-selectin, and monocyte chemoattractant protein-1 and further accelerates the formation of atherosclerotic plaques in aortic roots and increases the thickness of the coronary artery wall [27].

Inflammation has a central role in triggering acute coronary syndrome and is an underlying factor in the increased AMI risk with gastroenteritis [28]. Inflammation of the digestive tract may cause systemic inflammation and immune responses [29]. Studies have demonstrated that systemic inflammatory activity may directly influence coagulation pathways in cardiovascular diseases [13,14,28]. The inflammatory mediators C-reactive protein and interferon-gamma were found to enhance the expression of macrophage tissue factor levels that may contribute to the hypercoagulable state in coronary disease [14]. Inflammatory cells may infiltrate the coronary bed and plaques that have disrupted surfaces, and these cells produce cytokines, proteases, and coagulation factors, which increase endothelial damage, disrupt the fibrous cap, and initiate the formation of thrombi [30,31]. Moreover, platelets can be directly activated by systemic inflammation [32]. Severe inflammation induces a hyperdynamic cardiovascular response and disturbs hemodynamic homoeostasis; the effects of these complications include increased cardiac metabolic demands, decreased central blood pressure, and increased variations in systemic vascular resistance, which may alter myocardial metabolic balance and contribute further to the development of AMI [33].

Interestingly, our subgroup analysis showed that the increased risk of AMI after gastroenteritis was especially augmented in patients without ischemic heart disease and heart failure. However, our datasets lacked information about the subtypes of AMI. We considered that more aggressive monitoring and care provided as a result of the increased awareness of hemodynamic consequences of cardiovascular disease at diagnosis of gastroenteritis.

AMI as a result of gastroenteritis may be life-threatening if not identified and managed promptly. However, there have been no established guidelines for the prevention and treatment of AMI after acute gastroenteritis. We recommend regular cardiac screening of electrocardiograms with or without a cardiac enzyme test to identify possible myocardial infarction for cardiovascular high-risk patients experiencing severe gastroenteritis early [34]. Additionally, the pathogenesis of dehydration and the prothrombotic state necessary for the development of AMI suggest that adequate and timely fluid resuscitation and antithrombin therapy may prevent or ameliorate the myocardial ischemia induced by gastroenteritis [23,24,25,26,27,28,29]. Electrolyte and osmotic imbalance should be recognized early and corrected to avoid the increased production of thrombogenic molecules in gastroenteritis-related diarrhea [25,26,35].

There are limitations to our study, however. First, our data lack information about socioeconomic status, lifestyle factors, physical measures (e.g., blood pressure and electrocardiogram), biochemical laboratory measures (e.g., levels of cardiac enzyme, serum electrolytes, and confirmatory testing for pathogens of gastroenteritis), clinical data on detailed medication and fluid therapy, and clinical risk scores of acute coronary syndrome (such as the Thrombolysis In Myocardial Infarction scoring system) [36]. This information was not covered by health insurance databases. Second, subtypes of myocardial infarction were unknown in our study, such as type I or type II, and ST elevation or non-ST elevation myocardial infarction. Third, we cannot determine the exact etiology of gastroenteritis due to the unavailability of appropriate data. Fourth, our dataset did not include patients with gastroenteritis who did not seek conventional medical care due to mild symptoms. In addition, all hospitalized AMI patients in this study need to have positive findings of EKG, cardiac enzymes test, and cardiac catheterization according to the regulation from the Taiwan’s National Health Insurance. Nevertheless, we could not totally exclude the possibility of myocarditis in our AMI study subjects in this study. Finally, residual confounding is possible, although our analyses have adjusted for a variety of potential confounding factors.

In conclusion, gastroenteritis may act as a trigger for AMI and worsen post-AMI outcomes. Clinicians need to be aware of this severe complication of gastroenteritis. There is an urgent need for future studies on rehydration and antithrombin therapy to mitigate the AMI risk after severe gastroenteritis and accompanying dehydration.

## Figures and Tables

**Table 1:** Characteristics of AMI patients and controls (after propensity score matching 1:1).

	Controls (*N* = 103,584)		AMI (*N* = 103,584)		*p*-Value
Gastroenteritis	*n*	(%)	*n*	(%)	<0.0001
No	102,755	(99.2)	102,203	(98.7)	
Yes	829	(0.8)	1381	(1.3)	
Sex					1.0000
Female	25,306	(24.4)	25,306	(24.4)	
Male	78,278	(75.6)	78,278	(75.6)	
Age, years					1.0000
20–29	262	(0.3)	262	(0.3)	
30–39	2905	(2.8)	2905	(2.8)	
40–49	11,343	(11.0)	11,343	(11.0)	
50–59	21,580	(20.8)	21,580	(20.8)	
60–69	23,651	(22.8)	23,651	(22.8)	
70–79	21,470	(20.7)	21,470	(20.7)	
≥80	22,373	(21.6)	22,373	(21.6)	
Low income					1.0000
No	100,816	(97.3)	100,816	(97.3)	
Yes	2768	(2.7)	2768	(2.7)	
Number of hospitalizations					1.0000
0	81,385	(78.6)	81,385	(78.6)	
1	14,165	(13.7)	14,165	(13.7)	
2	4295	(4.2)	4295	(4.2)	
≥3	3739	(3.6)	3739	(3.6)	
Number of emergency visits					1.0000
0	64,931	(62.7)	64,931	(62.7)	
1	22,485	(21.7)	22,485	(21.7)	
2	8093	(7.8)	8093	(7.8)	
≥3	8075	(7.8)	8075	(7.8)	
Coexisting medical conditions					
Hypertension	38,972	(37.6)	38,972	(37.6)	1.0000
Diabetes	26,343	(25.4)	26,343	(25.4)	1.0000
Ischemic heart disease	21,810	(21.1)	21,810	(21.1)	1.0000
Mental disorders	15,774	(15.2)	15,774	(15.2)	1.0000
COPD	11,577	(11.2)	11,577	(11.2)	1.0000
Hyperlipidemia	6342	(6.1)	6342	(6.1)	1.0000
Heart failure	4655	(4.5)	4655	(4.5)	1.0000
Stroke	3737	(3.6)	3737	(3.6)	1.0000
Renal dialysis	2843	(2.7)	2843	(2.7)	1.0000
Liver cirrhosis	1372	(1.3)	1372	(1.3)	1.0000
Atrial fibrillation	687	(0.7)	687	(0.7)	1.0000

AMI: acute myocardial infarction; COPD, chronic obstructive pulmonary disease.

**Table 2:** Stratified analyses for the risk of AMI in patients with and without gastroenteritis (after propensity score matching).

		Controls		AMI		Risk of AMI	
		*n*	%	*n*	%	OR	(95% CI) ^†^
All	Gastroenteritis	829	(0.8)	1381	(1.3)	1.68	(1.54–1.83)
Female	Gastroenteritis	224	(0.9)	445	(1.8)	2.01	(1.71–2.36)
Male	Gastroenteritis	605	(0.8)	936	(1.2)	1.56	(1.40–1.72)
20–49 years	Gastroenteritis	103	(0.7)	154	(1.1)	1.50	(1.17–1.93)
50–59 years	Gastroenteritis	117	(0.5)	243	(1.1)	2.09	(1.68–2.61)
60–69 years	Gastroenteritis	198	(0.8)	294	(1.2)	1.49	(1.25–1.79)
70–79 years	Gastroenteritis	212	(1.0)	322	(1.5)	1.53	(1.28–1.82)
≥80 years	Gastroenteritis	199	(0.9)	368	(1.6)	1.86	(1.57–2.22)
0 medical condition	Gastroenteritis	158	(0.6)	277	(1.0)	1.76	(1.45–2.14)
1 medical condition	Gastroenteritis	284	(0.8)	501	(1.3)	1.78	(1.53–2.06)
2 medical conditions	Gastroenteritis	208	(0.8)	365	(1.5)	1.77	(1.49–2.10)
≥3 medical conditions	Gastroenteritis	179	(1.2)	238	(1.6)	1.34	(1.10–1.62)
0 hospitalization	Gastroenteritis	620	(0.8)	1071	(1.3)	1.74	(1.57–1.92)
1 hospitalization	Gastroenteritis	140	(1.0)	201	(1.4)	1.44	(1.16–1.79)
2 hospitalizations	Gastroenteritis	38	(0.9)	50	(1.2)	1.32	(0.86–2.02)
≥3 hospitalizations	Gastroenteritis	31	(0.8)	59	(1.6)	1.92	(1.24–2.98)
0 emergency visit	Gastroenteritis	415	(0.6)	728	(1.1)	1.76	(1.56–1.99)
1 emergency visit	Gastroenteritis	199	(0.9)	353	(1.6)	1.79	(1.50–2.13)
2 emergency visits	Gastroenteritis	104	(1.3)	156	(1.9)	1.51	(1.18–1.94)
≥3 emergency visits	Gastroenteritis	111	(1.4)	144	(1.8)	1.30	(1.02–1.67)
No IHD	Gastroenteritis	588	(0.7)	1076	(1.3)	1.84	(1.67–2.04)
IHD	Gastroenteritis	241	(1.1)	305	(1.4)	1.27	(1.07–1.50)
No stroke	Gastroenteritis	811	(0.8)	1343	(1.3)	1.67	(1.53–1.82)
Stroke	Gastroenteritis	18	(0.5)	38	(1.0)	2.15	(1.22–3.78)
No heart failure	Gastroenteritis	777	(0.8)	1319	(1.3)	1.71	(1.56–1.87)
Heart failure	Gastroenteritis	52	(1.1)	62	(1.3)	1.20	(0.82–1.73)

CI, confidence interval; OR, odds ratio; AMI: acute myocardial infarction; IHD, ischemic heart disease. ^†^ Adjusted for all covariates listed in Table 1.

**Table 3:** Characteristics of gastroenteritis in association with risk of AMI.

	Controls		AMI		Risk of AMI	
	*n*	%	*n*	%	OR	(95% CI) ^†^
No gastroenteritis hospitalization	103,574	(99.99)	103,559	(99.98)	1.00	(reference)
Gastroenteritis hospitalization	10	(0.01)	25	(0.02)	2.50	(1.20–5.21)

CI, confidence interval; OR, odds ratio. AMI: acute myocardial infarction. ^†^ Adjusted for all covariates listed in Table 1.

**Table 4:** Characteristics of AMI patients with and without gastroenteritis.

	No Gastroenteritis (*N* = 123,343)		Gastroenteritis (*N* = 1726)		*p*-Value
Sex	*n*	(%)	*n*	(%)	<0.0001
Female	33,162	(26.9)	612	(35.5)	
Male	90,181	(73.1)	1114	(64.5)	
Age, years					<0.0001
20–29	352	(0.3)	11	(0.6)	
30–39	3771	(3.1)	54	(3.1)	
40–49	13,325	(10.8)	138	(8.0)	
50–59	25,651	(20.8)	292	(16.9)	
60–69	28,807	(23.4)	394	(22.8)	
70–79	26,098	(21.2)	409	(23.7)	
≥80	25,339	(20.5)	428	(24.8)	
Low income					0.0785
No	118,700	(96.2)	1647	(95.4)	
Yes	4643	(3.8)	79	(4.6)	
Number of hospitalizations					0.0018
0	89,701	(72.7)	1214	(70.3)	
1	18,689	(15.2)	290	(16.8)	
2	7406	(6.0)	87	(5.0)	
≥3	7547	(6.1)	135	(7.8)	
Number of emergency visits					<0.0001
0	66,281	(53.7)	754	(43.7)	
1	28,639	(23.2)	440	(25.5)	
2	13,074	(10.6)	237	(13.7)	
≥3	15,349	(12.4)	295	(17.1)	
Coexisting medical conditions					
Hypertension	47,639	(38.6)	757	(43.9)	<0.0001
Diabetes	36,249	(29.4)	579	(33.6)	0.0002
Ischemic heart disease	36,890	(29.9)	551	(31.9)	0.0695
Mental disorders	19,746	(16.0)	360	(20.9)	<0.0001
COPD	14,768	(12.0)	254	(14.7)	0.0005
Heart failure	10,109	(8.2)	177	(10.3)	0.0020
Hyperlipidemia	8437	(6.8)	146	(8.5)	0.0083
Renal dialysis	8431	(6.8)	113	(6.6)	0.6371
Stroke	6368	(5.2)	78	(4.5)	0.2297
Liver cirrhosis	2333	(1.9)	44	(2.6)	0.0469
Atrial fibrillation	1623	(1.3)	31	(1.8)	0.0829

COPD, chronic obstructive pulmonary disease.

**Table 5:** Outcomes after AMI in patients with and without gastroenteritis.

	No Gastroenteritis (*N* = 123,343)		Gastroenteritis (*N* = 1726)		Outcome Risk	
	Events	%	Events	%	OR	(95% CI) ^†^
30-day in-hospital mortality	8185	6.6	152	8.8	1.28	(1.08–1.52)
ICU stay	108,251	87.8	1493	86.5	0.97	(0.84–1.12)
Medical expenditure, USD ^‡^	5531 ± 5115		5905 ± 6511		*p* = 0.0174	
Length of hospital stay, days ^‡^	7.7 ± 8.1		8.7 ± 8.6		*p* < 0.0001	

CI, confidence interval; OR, odds ratio; AMI: acute myocardial infarction. ^†^ Adjusted for all covariates listed in Table 1. ^‡^ Mean ± SD.

## Data Availability

The data underlying this study is from the Health andWelfare Data Science Center. Interested researchers can obtain the data through formal application to the Health andWelfare Data Science Center, Department of Statistics, Ministry of Health andWelfare, Taiwan. Under the regulations from the Health and Welfare Data Science Center, we have made the formal application (included application documents, study proposals, and ethics approval of the institutional review board) of the current insurance data. The authors of the present study had no special access privileges in accessing the data which other interested researchers would not have.

## Supplementary Materials

The following supporting information can be downloaded at: https://www.mdpi.com/2077-0383/11/5/1341/s1, Table S1: Characteristics of AMI patients and controls (frequency matching by age and sex, case-onctrol ratio = 1:4). Table S2: The sensitivity analysis for the risk of AMI associated with gastroenteritis.

## Abbreviations

AMI = acute myocardial infarction; ICD-9-CM = International Classification of Diseases, 9th Revision, Clinical Modification; CI = confidence interval; OR = odds ratio.
