# Supplementary material for: Risk of Acute Myocardial Infarction in Patients with Gastroenteritis: A Nationwide Case-Control Study
Source: J Clin Med. 2022 Feb 28;11(5):1341. doi: 10.3390/jcm11051341 (PMC8911228; doi:10.3390/jcm11051341)
Supplement: Supplementary file 1 [file jcm-11-01341-s001.zip › jcm-1581807-supplementary.pdf]

Table S1. Characteristics of AMI patients and controls (frequency matching by age and sex, case-onctrol ratio = 1:4)

	Controls (N=500,276)		AMI (N=125,069)		*p*-value
Gastroenteritis	n	(%)	n	(%)	<0.0001
No	496578	(99.3)	123343	(98.6)	
Yes	3698	(0.7)	1726	(1.4)	
Sex					1.0000
Female	135096	(27.0)	33774	(27.0)	
Male	365180	(73.0)	91295	(73.0)	
Age, years					1.0000
20-29	1452	(0.3)	363	(0.3)	
30-39	15300	(3.1)	3825	(3.1)	
40-49	53852	(10.8)	13463	(10.8)	
50-59	103772	(20.7)	25943	(20.7)	
60-69	116804	(23.4)	29201	(23.4)	
70-79	106028	(21.2)	26507	(21.2)	
≥80	103068	(20.6)	25767	(20.6)	
Low income					<0.0001
No	483384	(96.6)	120347	(96.2)	
Yes	16892	(3.4)	4722	(3.8)	
Number of hospitalizations					<0.0001
0	415409	(83.0)	90915	(72.7)	
1	53323	(10.7)	18979	(15.2)	
2	16391	(3.3)	7493	(6.0)	
≥ 3	15153	(3.0)	7682	(6.1)	
Number of emergency visits					<0.0001
0	380521	(76.1)	67035	(53.6)	
1	74030	(14.8)	29079	(23.3)	
2	24657	(4.9)	13311	(10.6)	
≥ 3	21068	(4.2)	15644	(12.5)	
Coexisting medical conditions					
Hypertension	177043	(35.4)	48396	(38.7)	<0.0001
Diabetes	92460	(18.5)	36828	(29.5)	<0.0001
Hyperlipidemia	42888	(8.6)	8583	(6.9)	<0.0001
Mental disorders	94022	(18.8)	20106	(16.1)	<0.0001
Ischemic heart disease	49200	(9.8)	37441	(29.9)	<0.0001
Stroke	15233	(3.0)	6446	(5.2)	<0.0001
Atrial fibrillation	4620	(0.9)	1654	(1.3)	<0.0001
COPD	62320	(12.5)	15022	(12.0)	<0.0001
Liver cirrhosis	14281	(2.9)	2377	(1.9)	<0.0001
Renal dialysis	6356	(1.3)	8544	(6.8)	<0.0001
Heart failure	11997	(2.4)	10286	(8.2)	<0.0001

AMI: acute myocardial infarction; COPD, chronic obstructive pulmonary disease.

Table S2. The sensitivity analysis for the risk of AMI associated with gastroenteritis

	Controls (n=13620)		AMI patients (n=7646)†		Risk of AMI
	n	%	n	%	OR (95% CI)*
No gastroenteritis	13550	99.5	7538	98.6	1.00 (reference)
Gastroenteritis	70	0.5	108	1.4	2.89 (2.13-3.92)

AMI, acute myocardial infarction; CI, confidence interval; OR, odds ratio.

* Adjusted for all covariates listed in Table 1.

†Defined as patients with AMI admission who had received percutaneous coronary intervention or coronary artery bypass graft.
